# DEMANet: A dehazing enhanced multi-branch attention network for remote sensing images

**DOI:** 10.1371/journal.pone.0354121

**Published:** 2026-09-08

**Authors:** Peixue Liu, Shu Liu, Pengfei He, Guoheng Wang

**Affiliations:** 1 Qingdao Huanghai University, Qingdao, Shandong, China; 2 School of Physics and Electronic Information, Yantai University, Yantai, Shandong, China; 3 Shandong Data Open Innovation Application Laboratory of Smart grid Advanced Technology, Yantai University, Yantai, Shandong, China; 4 Yantai Dongfang Wisdom Electric Company Limited, Yantai, Shandong, China; Shanghai Jiao Tong University, CHINA

## Abstract

Haze represents a key constraint on the application of optical remote sensing imagery. It not only impairs visual quality but also lowers the accuracy of remote sensing interpretation tasks such as classification and change detection. To tackle this issue, we present a Dehazing Enhanced Multi-branch Attention Network (DEMANet) for effective remote sensing image dehazing. The network adopts a U-Net-like structure with three hierarchical downsampling stages to implement progressive feature extraction from shallow to deep layers. Shallow and middle layers use a residual dual-path module to enhance local detailed features via a main-auxiliary dual-branch structure. Deep layers employ a dual-attention module with a two-layer attention mechanism to break the limitation of local receptive fields in traditional convolutions and accurately capture global semantics and haze features. A cross-stage feature interaction module embeds a haze-guided mechanism to locate haze regions based on edge and color differences, enabling cross-stage feature alignment and interaction between encoding and decoding. This reduces information loss during upsampling and improves detail preservation and dehazing performance. Experiments conducted on widely used public remote sensing datasets demonstrate that our innovative approach outperforms existing algorithms in haze removal, while simultaneously preserving intricate image details and color fidelity.

## 1 Introduction

Spaceborne optical imaging sensors frequently encounter challenges when capturing images from great distances, as atmospheric elements like fog, haze, and clouds tend to throw a wrench in the works, causing significant degradation in remote sensing images (RSI) quality. This causes the loss of complex detailed information, blurred texture clarity and reduced contrast, significantly increasing the difficulty of subsequent data processing and analysis [1]. Therefore, improving the quality of blurred RSI is a crucial preprocessing task for practical applications in various fields.

Restoring visibility in hazy images through RSI techniques stands as a critical challenge in foundational computer vision, with the primary objective of resurrecting crisp visuals from the visual chaos created by atmospheric interference and light diffusion. Over the past few years, numerous image dehazing methods have been proposed for RSI, which can be mainly categorized into physics-based methods and deep learning-based methods. Physics-based dehazing techniques restore clear images using physical models by estimating parameters such as atmospheric light and transmission rate. However, such algorithms are somewhat limited in development due to their heavy reliance on manually estimated intermediate variables and separate processing of the dehazing procedure [2]. Deep learning has been turning heads in computer vision lately, giving rise to a whole new crop of image dehazing methods that leverage convolutional neural networks (CNNs) [3]. Rather than relying on the convoluted preprocessing and postprocessing steps characteristic of conventional methods, these approaches leverage neural networks to crack the code on the nonlinear relationship between hazy and clear images, effectively cutting through the fog. Nevertheless, they still suffer from drawbacks including inefficient convolution operations, insufficient feature extraction, and unsatisfactory performance on non-uniform hazy images.

To overcome these limitations, we introduce an innovative DEMANet, which effectively improves the overall dehazing performance compared with previous methods. DEMANet adopts a U-Net-like encoder-decoder as its overall framework. By means of symmetric downsampling and upsampling operations, the network can realize multi-scale feature extraction and reconstruction. To mitigate the issue of detail loss in remote sensing images, we embed different innovative modules at various feature levels to optimize the network. Specifically, the novel Residual Dual Path Block (RDPB) is introduced in the shallow and middle feature processing stages. Based on optimized residual convolution, this module adopts a dual-branch structure with two-level residual connections, which effectively avoids detail blurring and reduces feature redundancy. The novel Feature Interaction Block (FIB) is employed for cross-stage feature fusion. By combining explicit haze region modeling and an attention mechanism, this block achieves accurate feature interaction and fusion, and effectively alleviates information loss during upsampling, inaccurate haze localization, and misalignment of features. The Dual Attention Block (DAB) [4] is embedded in the deep layers to model the correlations between global and local features via a dual-attention mechanism, enhancing the capability of capturing features in complex haze regions. The primary contributions of this work can be summarized as follows:

(1) To address detail degradation and feature redundancy in RSI dehazing, this paper proposes a Residual Dual Path Block (RDPB). Unlike traditional single-branch structures, RDPB adopts a dual-path architecture. It integrates standard convolution for semantic extraction, lightweight DEConv [5] and GSConv [6] for detail refinement, and dual residual fusion to effectively retain complementary features.
(2) To address information loss in upsampling, inaccurate haze localization, and cross-stage alignment issues in RSI dehazing, we propose the Feature Interaction Block (FIB). It integrates haze region guidance and attention for precise decoder-skip connection fusion, enabling adaptive alignment and multi-scale feature enhancement in complex hazy scenarios.
(3) To address insufficient deep global semantic exploitation and inaccurate complex haze feature capture in remote sensing image dehazing, we propose the Dual Attention Block (DAB). It innovatively combines serial CSAM and parallel PAM. CSAM locates key haze features, while PAM integrates multi-dimensional attention information to mine global semantic and haze distribution features, improving detail retention and model robustness.
(4) Breaking the limitation of traditional fixed-weight loss functions, we propose a heteroscedastic uncertainty-based adaptive weighted fusion loss. Combined with contrastive loss, it forms a composite loss function for adaptive feature optimization. Experimental results on StateHaze1k and RRSHID datasets show that the proposed DEMANet achieves superior dehazing performance and detail preservation over state-of-the-art methods.

This paper continues with the following structure. Section 2 surveys existing literature concerning image dehazing research. Section 3 describes DEMANet's architecture and the detailed structure and function of its components. Section 4 details comparative and ablation studies assessing DEMANet's dehazing efficacy. Section 5 concludes this paper.

## 2. Related work

### 2.1 Physics‑based image dehazing method

Physics-based methods typically develop an atmospheric scattering model [7] to characterize the degradation factors affecting scene imagery, subsequently reconstructing the original scene by leveraging this model under specific predetermined conditions. The atmospheric scattering model can be formulated as follows:


$$\begin{array}{c} I\left(x\right)=J\left(x\right)t\left(x\right)+A\left(1-t\left(x\right)\right) \end{array}$$ (1)

Where I(x) denotes the hazy observation image, A denotes the atmospheric light, and t(x) denotes the transmission map. Such methods rely on manually designed priors to estimate global atmospheric light and transmission maps, thereby restoring haze-free images J(x). The restoration formula can be derived by reformulating Equation (1):


$$\begin{array}{c} J\left(x\right)=\frac{\text{1}}{t\left(x\right)}I\left(x\right)-A\frac{\text{1}}{t\left(x\right)}+A \end{array}$$ (2)

Back in 2009, He et al. [8] introduced the groundbreaking dehazing technique that built upon the Dark Channel Prior (DCP). This approach marked a significant leap by merging the DCP with atmospheric scattering modeling while incorporating a prior rooted in statistical assumptions. On another front, Fattal [9] put forward a single-image dehazing approach that harnessed the “color-lines” regularity commonly observed in natural imagery. By deriving the local hazy image formation model, robustly extracting color lines and estimating the transmission rate using the Random Sample Consensus algorithm, and implementing interpolation and regularization of the transmission map with an enhanced Gaussian-Markov random field with long-range connections, more accurate transmission estimation and dehazing performance were achieved. Zhu et al. [10] pioneered the Color Attenuation Prior (CAP), an approach that examines fluctuations in pixel brightness and saturation throughout an image. They developed a linear framework grounded in CAP that proves adept at reconstructing depth data. Li [11] put forward a technique dubbed “RDT” aimed at eliminating nighttime image noise by leveraging Retinex theory and Taylor series expansion. The final step in producing a haze-free image involves utilizing both the atmospheric light image and the latent haze-free counterpart during image fusion and color transformation processes. Liu et al. [12] proposed the Inverted Haze Density Correction Prior (IHDCP), a physics-based prior that models image transmission via pixel-wise gamma correction on the inverted haze density map. They integrated IHDCP into the atmospheric scattering model and derived an efficient solution using Taylor expansion and boundary constraints for accurate and fast single image dehazing. However, physics-based dehazing methods rely on specific manually designed priors, which only hold under most natural scenarios and cannot cover the complexity of all hazy conditions, resulting in poor generalization ability under non-ideal situations.

### 2.2 Deep learning-based dehazing methods

Over the past few years, as deep learning technology has made great strides, approaches to dehazing images using these advanced techniques have taken center stage in image dehazing research. These cutting-edge methodologies generally fall into two main buckets. The initial group utilizes neural networks to determine atmospheric scattering model parameters, followed by reconstructing the clear image by reversing through this established physical framework. Cai et al. [13] first constructed DehazeNet, a trainable deep neural network. DehazeNet is utilized to assess the transmission rate of the input image. Afterwards, the global atmospheric light value is evaluated with the help of some image prior information. In the end, the haze-free image is retrieved on the basis of the atmospheric scattering formula. The advantage of such methods is that they preserve the interpretability of the physical model, and the parameter estimation results are more consistent with the actual imaging laws. However, they have obvious limitations: there are cumulative errors in parameter estimation, which lead to color distortion, haze residue and other problems in the recovered haze-free images. Moreover, parameter estimation and haze-free image restoration are independent of each other, making it impossible to achieve collaborative optimization of the two processes. In addition to DehazeNet, subsequent scholars have conducted improved research on such methods to further enhance the accuracy and robustness of parameter estimation. For example, Ren et al. [14] proposed Multi-scale Convolutional Neural Networks (MSCNN). This network managed to nail down both transmission rate and atmospheric light value estimates simultaneously by leveraging a multi-scale convolutional architecture. This approach beefed up the reliability of parameter estimation by tapping into features that cover various receptive fields, making the whole system more robust against different conditions.

The other category encompasses those end-to-end dehazing algorithms that have gained considerable traction in the field. These techniques take a fog-affected picture as their starting point and spit out a clear image without any intermediate steps. What sets them apart is that they don't lean on physical models to get the job done; instead, they can autonomously learn the degradation rules of hazy images through training on large-scale datasets, effectively solving the problem of cumulative error. This has become the most widely used dehazing approach at present. Li et al. [15] proposed an all-in-one dehazing network (AOD-Net). It first reformulates the atmospheric scattering model to combine the two originally estimated unknown parameters into one parameter, and then directly outputs a clear image based on the revised atmospheric scattering formula. Specifically, the parameters t(x) and A are integrated and replaced by K(x). Equation (2) can be rewritten as follows:


$$\begin{array}{c} J\left(x\right)=K\left(x\right)I\left(x\right)-K\left(x\right)+b \end{array}$$ (3)

where $K\left(x\right)=\frac{\frac{\text{1}}{t\left(x\right)}\left(I\left(x\right)-A\right)+(A-b)}{I\left(x\right)-1}$, b is a constant bias term with a default value of 1.

Engin et al. [16] employed a cycle generative adversarial network to remove haze from images. Unlike conventional methods, this framework does not depend on paired training data; it directly takes hazy images as input and optimizes network learning by combining cycle‑consistency loss and perceptual loss, thus resulting in markedly enhanced image reconstruction quality. Lu et al. [17] proposed a novel hybrid building-block dehazing network named MixDehazeNet in 2023. Taking U-Net as the backbone, they designed the Multi-Scale Parallel Large Convolution Kernel module and Enhanced Parallel Attention module with large receptive field and multi-scale characteristics, solving the problems of neglected multi-scale properties and insufficient handling of non-uniform haze distribution in image dehazing tasks. In 2024, Wen et al. [18] proposed RSHazeNet, a lightweight encoder-decoder framework dedicated to remote sensing image dehazing, which preserves dehazing efficacy while optimizing processing speed for high-resolution RSI dehazing applications. In addition, Transformer models are now extensively utilized for visual applications including image dehazing. For example, in 2023, Qiu et al. [19] proposed a multi-branch optimized Transformer architecture for haze removal in images, which reduces self-attention's computational complexity to linear complexity by Taylor expansion and corrects Taylor expansion errors with a multi-scale attention refinement module. It also shows favorable generalization ability in tasks of snow removal and rain removal besides dehazing. Song et al. [20] proposed DehazeFormer, a novel paradigm for image dehazing. The method delivers exceptional dehazing results across both synthetic datasets and demonstrates powerful dehazing capabilities when applied to extensive real-world remote sensing imagery. In recent years, several representative approaches have been developed for nighttime image dehazing. For instance, Liu et al. [21] proposed NightHazeFormer, a Transformer-based architecture for single nighttime image dehazing, which employs a two-stage pipeline consisting of supervised pre-training and semi-supervised fine-tuning to effectively alleviate multiple degradations, including glow, blur, noise, and color distortion. Liu et al. [22] proposed a novel Variational Nighttime Dehazing framework using Hybrid Regularization, which achieves nighttime image glow elimination, haze removal, brightness improvement and noise reduction, and is applicable to the restoration of low-light, sandstorm, underwater and other degraded images.

## 3 Proposed method

We begin this section by presenting DEMANet's structure, then describe each component module of DEMANet in detail, and finally elaborate on the loss function used for training.

### 3.1 Overall architecture

**Fig 1:**
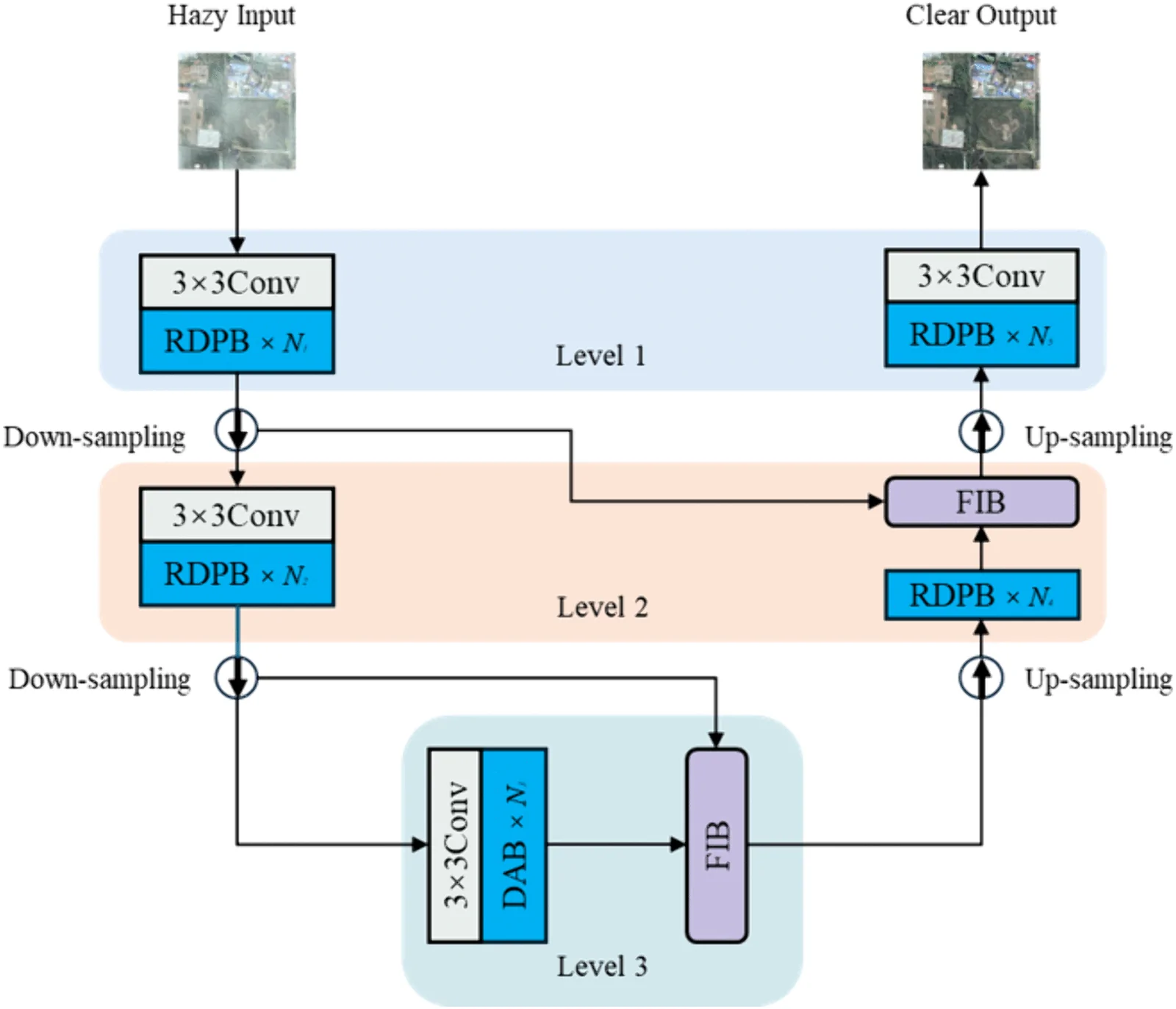
DEMANet structure.

DEMANet integrates multi-scale feature enhancement modules and a feature fusion mechanism, thereby realizing an end-to-end mapping from the input image to the restored image. Fig 1 provides a visual overview of the complete architectural framework. The network is divided into three feature processing hierarchies according to downsampling levels. Residual Dual Path Block (RDPB) is adopted in the shallow and middle layers, while Dual Attention Block (DAB) is used in the deep layer to strengthen global feature extraction. Feature Interaction Block (FIB) is applied to achieve cross-hierarchy feature fusion and improve feature complementarity.

### 3.2 Residual dual path block

**Fig 2:**
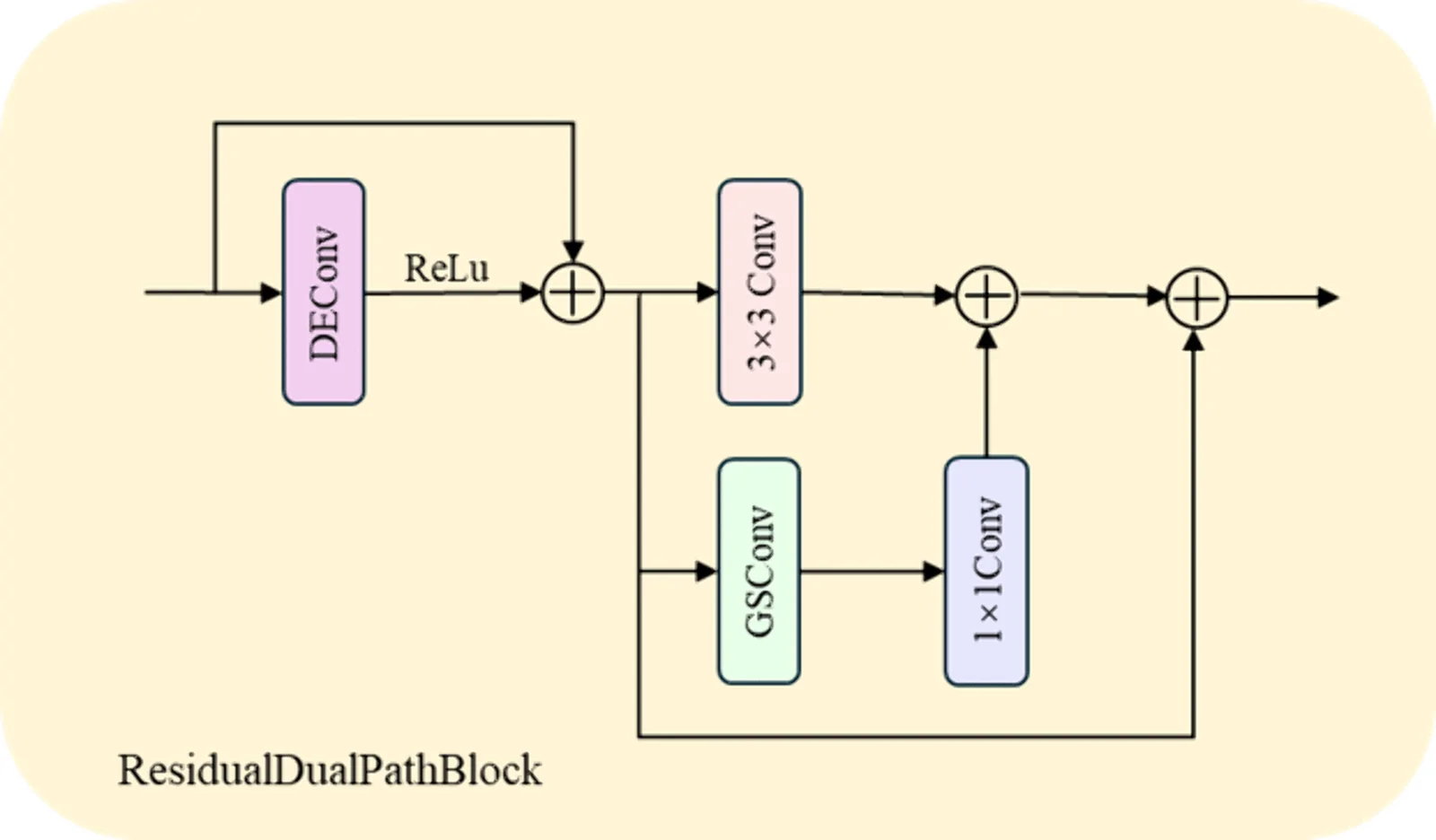
Residual dual path block structure.

The Residual Dual Path Block (RDPB) consists of an initial residual branch using DEConv, a main branch with standard convolution, an auxiliary branch using GSConv (Group Sparse Convolution), and a dual residual fusion mechanism. It serves as the core basic module for shallow and middle feature extraction in the dehazing network. Fig 2 illustrates RDPB's structure.

In RDPB, the DEConv initial residual branch performs basic feature extraction through the combination of convolution and activation while keeping the feature dimension unchanged. Through residual connection, it maintains the input image's original textural details, effectively alleviating the gradient vanishing problem of shallow and middle-layer features in the dehazing task. The standard convolution main branch takes standard convolution as the core, which helps capture the local correlation features between hazy and clear regions in the image. The GSConv auxiliary branch leverages the lightweight property of group sparse convolution to reduce computational complexity while enhancing the ability to capture multi-scale details, especially showing remarkable advantages in extracting fine textures in regions with uneven haze density. Finally, the module adopts a dual residual fusion strategy: it first fuses features from the main and auxiliary branches, then conducts a second fusion with the original residual features. This achieves deep complementarity between local features and multi-scale details. It not only significantly enhances DEMANet's capacity for capturing valuable shallow and medium-level features, but also effectively suppresses detail loss and artifacts in the dehazed image.The specific formulation is as follows:


$$\begin{array}{c} \begin{array}{c} \text{Y}={\text{Conv}}_{k\times k}(ReLU(\text{DEConv}\left(X\right))+X)+\\ \omega \cdot {\text{Conv}}_{1\times 1}\left(\text{GSConv}\left(ReLU\left(\text{DEConv}\left(X\right)\right)+X\right)\right)+ReLU\left(\text{DEConv}\left(X\right)\right)+X \end{array} \end{array}$$ (4)

Where X denotes the module's input feature map;Y denotes the output feature map; ${\text{Conv}}_{k\times k}(\cdot )$ signifies the standard convolution with kernel size k × k; ${\text{Conv}}_{1\times 1}(\cdot )$ represents the pointwise convolution with kernel size 1 × 1, which achieves channel recovery and feature fusion; DEConv(·)denotes the detail-enhanced convolution operation; GSConv(·) denotes the group sparse convolution operation; ReLU(·) is the ReLU activation function that enhances the nonlinear representation of features; ω is a learnable weight parameter with an initial value of 0.1, used to balance the feature weight of the auxiliary branch.

### 3.3 Dual attention block

**Fig 3:**
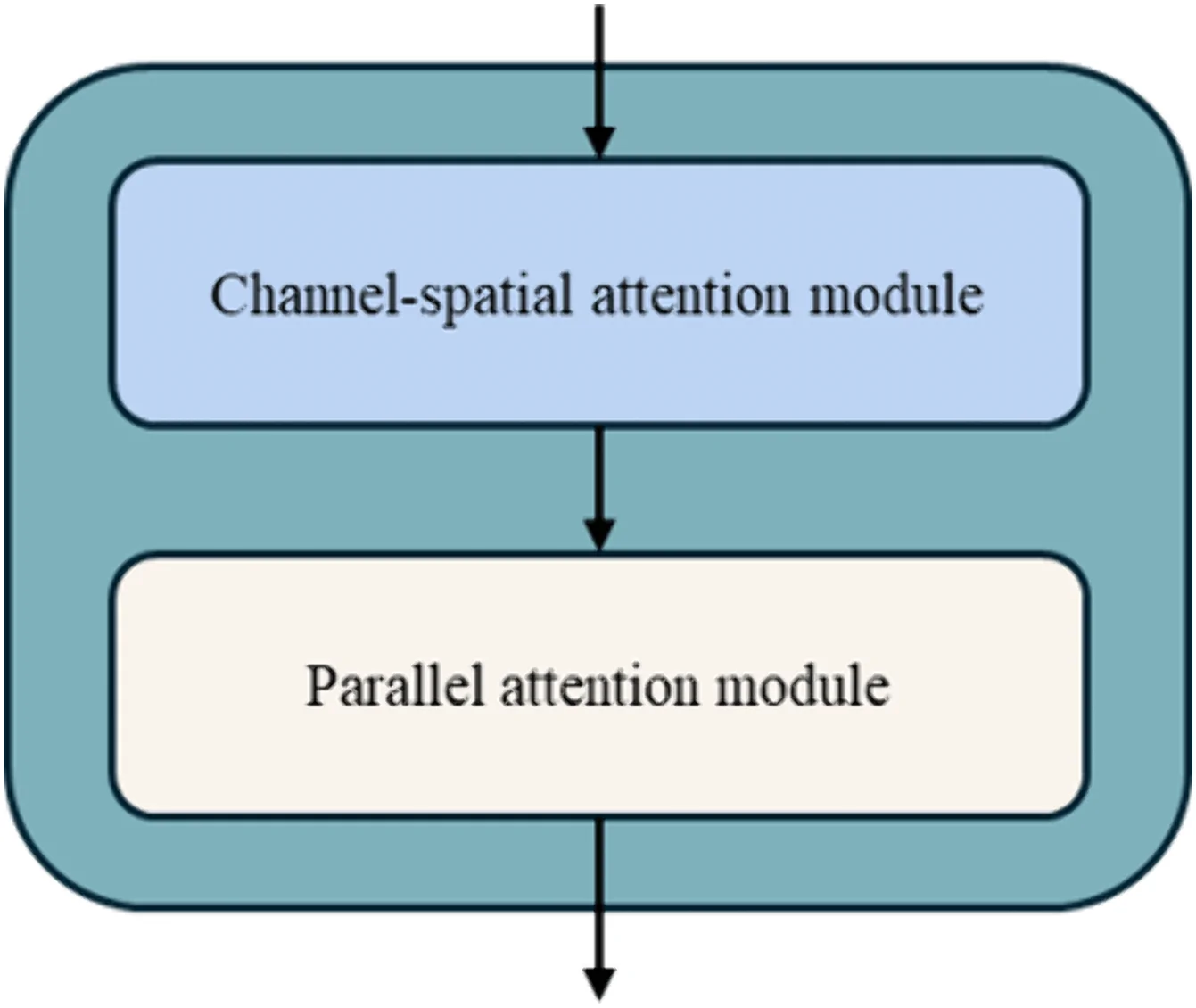
Dual attention block structure.

DualAttentionBlock (DAB) consists of two serially connected modules: CSAM and PAM. Its structure is shown in Fig 3. In the first stage, the CSAM integrates channel and spatial information to filter haze features and quickly locate haze distribution regions. In the second stage, the PAM captures haze features more accurately while preserving image details better.

**Fig 4:**
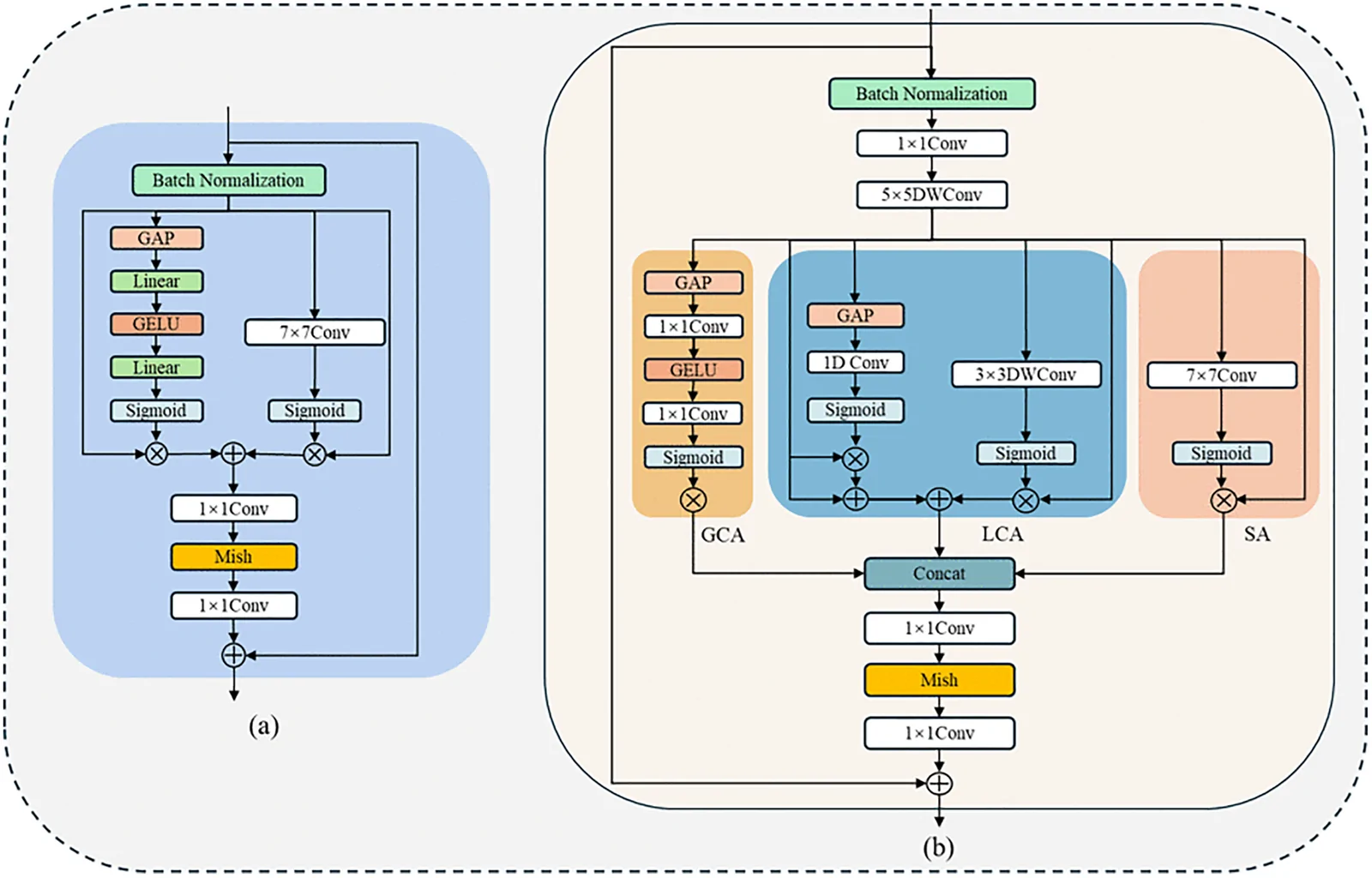
(a) illustrates the CSAM structure. (b) illustrates the PAM structure.

As illustrated in Fig 4(a), CSAM is designed to simultaneously capture channel-wise and spatial dependencies in the initial stage, thus achieving haze feature filtering. The PAM mainly consists of three attention modules, as shown in Fig 4(b). Global Channel Attention (GCA) uses Global Average Pooling (GAP) to obtain global compressed information per channel, then generates feature maps through two 1 × 1 convolutions to extract global cross-channel information. For Local Channel Attention (LCA), its interaction branch utilizes GAP to capture channel-wise global information, and then captures interaction information between local channels using 1D convolution (1D Conv). By employing 3 × 3 depthwise separable convolutions, the local channel branch both generates and extracts local information unique to each channel. This module integrates the information obtained from the two branches and effectively retains the channel information. Spatial Attention (SA) generates feature maps using wide-range spatial information produced by 7 × 7 large-kernel convolution, so as to better preserve image details.

### 3.4 Feature interaction block

**Fig 5:**
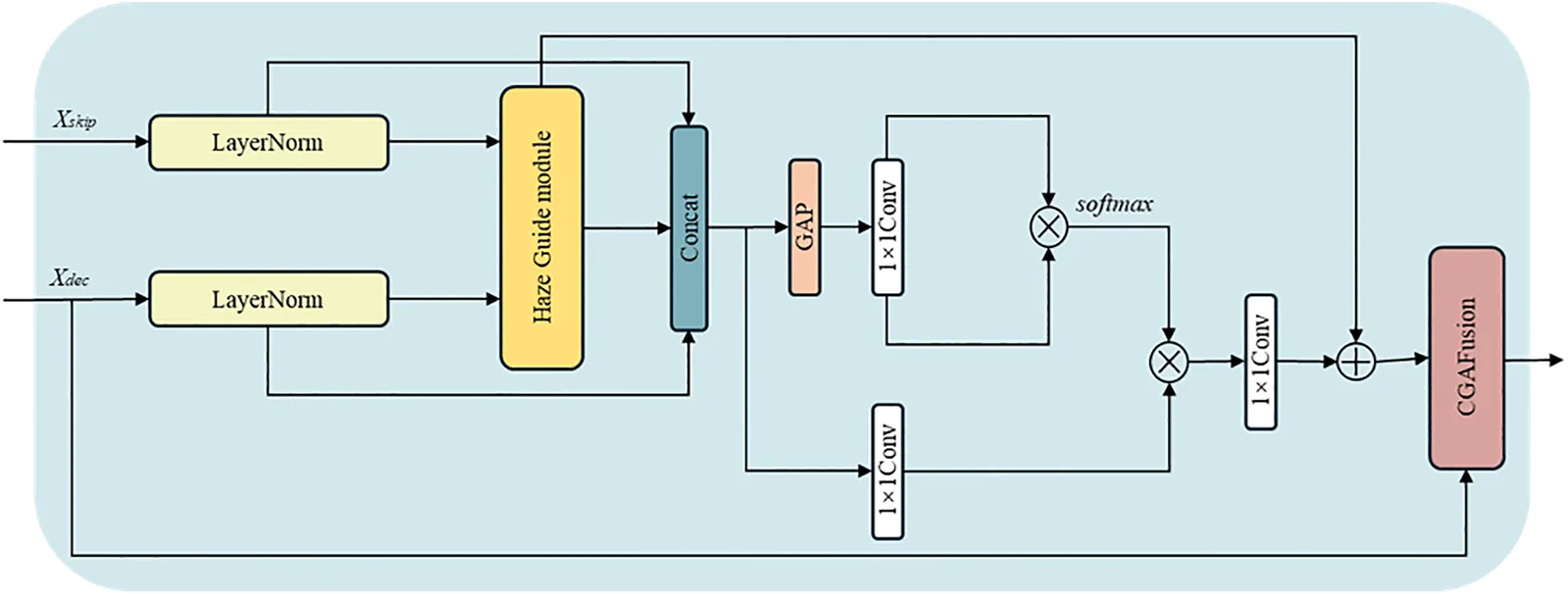
Feature Interaction Block structure.

To address the problems of inaccurate haze region location, insufficient multi-scale feature interaction, and severe detail loss existing in traditional feature fusion modules in dehazing tasks, this paper designs a haze-guided feature interaction block (FIB) as the core component for feature fusion in DEMANet, illustrated in Fig 5. Specifically, the proposed haze-guided mechanism takes the edge texture and color distribution difference of haze regions as the guidance signal to construct a dedicated haze feature mask. Then, the haze-guided feature mask is integrated into the QKV attention calculation to form an attention interaction mode between cross-stage features and haze guidance. In addition, the module introduces Content-Guided Attention Fusion (CGAFusion) [5], which fuses channel-wise, spatial, and pixel-level attention mechanisms to effectively blend attention-augmented features with the original decoding features.

According to the feature differences between hazy and clear regions in dehazing tasks, the haze-guided module first calculates the edge difference and brightness difference between the decoder branch features and skip branch features respectively. Then, a 3 × 3 convolution layer combines the preceding difference features to create the haze-guided feature, which helps to accurately locate the hazy regions in the image. The generated guidance feature can also effectively enhance the ability of the subsequent attention module to interact and fuse haze features, compensate for the feature misalignment between the decoder branch and skip branch caused by downsampling and upsampling, and strengthen the feature complementarity of hazy regions. The overall formulation of the haze-guided module is as follows:


$$\begin{array}{cc} & \text{G}\left(X_{dec},X_{skip}\right)={\text{Conv}}_{3\times 3}(\text{Concat}(|{𝔼}_{c}\left(|X_{dec}-P_{avg}\left(X_{dec}\right)|\right)-\\ & {𝔼}_{c}\left(|X_{skip}-P_{avg}\left(X_{skip}\right)|\right)\left|,\right|{𝔼}_{c}\left(X_{dec}\right)-{𝔼}_{c}\left(X_{skip}\right)|)) \end{array}$$ (5)

Where $X_{dec}$ and $X_{skip}$ are the layer-normalized decoder features and skip connection features respectively; $P_{avg}(\cdot )$ denotes the 3 × 3 average pooling operation with padding 1 and stride 1; |·| denotes element-wise absolute value; ${𝔼}_{c}\left[\cdot \right]$ denotes the channel-wise mean operation to aggregate multi-channel features into single-channel features. By calculating the absolute difference, the edge information difference and color brightness information difference between the decoder features and skip connection features can be effectively measured, and then the 3 × 3 convolution is used to fuse the above two types of difference features to finally obtain the haze-guided feature $\text{G}\in {\mathbb{R}}^{B\times 1\times H\times W}$.

### 3.5 Loss function

In this paper, the proposed method is trained using a hybrid loss function composed of the adaptive weighted fusion loss function ${ℒ}_{UMS}$ based on heteroscedastic uncertainty and the contrastive loss function ${ℒ}_{CR}$.

#### 3.5.1 Adaptive weighted fusion loss function based on heteroscedastic uncertainty.

The adaptive weighted fusion loss function based on heteroscedastic uncertainty fuses the pixel-level MSE loss and the structure-aware SSIM loss. The model learns the optimizable parameters $\sigma_{mse}$ and $\sigma_{ssim}$ to realize dynamic weight assignment for each loss term, where the weights are inversely proportional to the square of uncertainty. A warm-up regularization term is introduced to avoid parameter divergence, and gradient-free scale normalization is performed on the weighted loss to balance the numerical magnitude. All initial parameters are set to 1 to start training with equal weights, and no manual parameter tuning is required in the subsequent training process. The specific formulation is as follows.

(1) The basic loss terms are as follows:

$$\begin{array}{c} {ℒ}_{MSE}=\text{MSE}\left(\hat{I},I\right) \end{array}$$ (6)

$$\begin{array}{c} {ℒ}_{SSIM}=1-𝔼\left[\text{SSIM}\left(\hat{I},I\right)\right] \end{array}$$ (7)

Where ${ℒ}_{MSE}$ is the pixel-level mean squared error loss, ${ℒ}_{SSIM}$ is the structural similarity loss, 𝔼[·] denotes the average of SSIM values over all images in the batch, $\hat{I}$ denotes the predicted image, while I represents the ground-truth reference image.

(2) Uncertainty-weighted fusion loss:

$$\begin{array}{c} {ℒ}_{w}=\frac{{ℒ}_{MSE}}{2\sigma_{mse}^{\text{2}}}+\frac{{ℒ}_{SSIM}}{2\sigma_{ssim}^{\text{2}}} \end{array}$$ (8)

where $\sigma_{mse}$ and $\sigma_{ssim}$ are the learnable heteroscedastic uncertainty parameters.
(3) Dynamic regularization term:

$$\begin{array}{c} \lambda_{reg}\left(epoch\right)=\left\{ \begin{array}{cc} @ll@0.01\cdot \frac{epoch+1}{10}, & epoch<10,\\ 0.01, & epoch\geq 10 \end{array}\right. \end{array}$$ (9)

$$\begin{array}{c} {ℒ}_{reg}=\lambda_{reg}\cdot log\left(\sigma_{mse}\sigma_{ssim}\right) \end{array}$$ (10)

where $\lambda_{reg}$ denotes the regularization coefficient and epoch represents the current training epoch.
(4) Scale-normalize total loss:

$$\begin{array}{c} {ℒ}_{UMS}=\frac{{ℒ}_{w}}{({ℒ}_{w}{)}_{\text{detach}}}+{ℒ}_{reg} \end{array}$$ (11)

where ${\left(\cdot \right)}_{\text{detach}}$ denotes stopping the gradient backpropagation.

#### 3.5.2 Contrastive loss function.

The contrastive loss function ${ℒ}_{CR}$ is defined as follows:


$$\begin{array}{c} {ℒ}_{CR}=\sum_{k=1}^{\text{5}}\omega_{k}\cdot \frac{∥V_{k}\left(\hat{I}\right)-V_{k}\left(I\right){∥}_{\text{1}}}{∥V_{k}\left(\hat{I}\right)-V_{k}\left(I_{H}\right){∥}_{\text{1}}+10^{-7}},\text{\textbackslash{}hspace\{1em}\}\omega_{k}=\left\{\frac{\text{1}}{32},\frac{\text{1}}{16},\frac{\text{1}}{\text{8}},\frac{\text{1}}{\text{4}},1\right\} \end{array}$$ (12)

where $\hat{I}$ denotes the predicted image, I denotes the ground-truth reference image, $I_{H}$ denotes the hazy input image, $V_{k}\left(\cdot \right)$ represents the feature map from the k-th layer of the pre-trained VGG-19 network, $\omega_{k}$ is the weight coefficient of each feature layer, $∥\cdot {∥}_{\text{1}}$ denotes the L1 loss, and $10^{-7}$ serves as a stability factor to prevent division by zero.

#### 3.5.3 Total loss function.

By aggregating all individual loss terms, the total loss function is formulated as follows:


$$\begin{array}{c} {ℒ}_{\text{total}}=\lambda_{\text{1}}\cdot {ℒ}_{UMS}+\lambda_{\text{2}}\cdot {ℒ}_{CR} \end{array}$$ (13)

where $\lambda_{\text{1}}=1.0\text{,}\lambda_{\text{2}}=0.1$ are the weighting coefficients of the total loss.

## 4 Experiment

In this section, we first summarize the datasets used, experimental details, and evaluation metrics. We then carry out extensive comparison experiments with other advanced single-image dehazing techniques. Lastly, ablation studies are conducted to systematically evaluate the contribution of each module in DEMANet.

### 4.1 Datasets and implementation details

To evaluate DEMANet, we conducted experiments on the public remote sensing datasets SateHaze1k [23] and RRSHID-Moderate [24].

SateHaze1k consists of three sub-datasets: SateHaze1k-Thin, SateHaze1k-Moderate, and SateHaze1k-Thick. These three sub-datasets contain remote sensing images affected by thin, moderate, and thick haze levels respectively, along with their corresponding clear images. In each sub-dataset, we allocated 320 image pairs for training and 45 for testing.

RRSHID is divided into three subsets according to haze density: RRSHID-Thin, RRSHID-Moderate, and RRSHID-Thick. Among them, the RRSHID-Moderate subset is the largest one, containing 1526 pairs. We set 1220 pairs of images as the training set and 152 pairs as the test set.

We used the ADAM optimizer [25], and set β1, β2, ε to the default values of 0.9, 0.999, 1e-8, respectively. In addition, the initial learning rate was set to 4e-4, and the batch size was set to 8. The learning rate was adjusted from the initial value to 1e-6 using the cosine annealing strategy [26]. For data augmentation, random rotations of 90°, 180°, or 270° were adopted, and the input images were randomly cropped to 256 × 256. Experiments executed on an NVIDIA RTX 4090 GPU with PyTorch 2.0.1.

### 4.2 Evaluation metrics

The peak signal-to-noise ratio (PSNR), structural similarity index measure (SSIM) and CIE Delta E 2000(CIEDE) were selected as the evaluation metrics for algorithm performance. The evaluation results of the proposed algorithm were analyzed and compared with those of related methods in this study.

A higher PSNR value indicates lower distortion and higher pixel fidelity in the restored image. PSNR is defined as:


$$\begin{array}{c} \text{PSNR}=10\times log\left(\frac{{\text{Max}}_{I}^{\text{2}}}{\text{MSE}}\right) \end{array}$$ (14)

where ${\text{Max}}_{I}^{\text{2}}$ denotes the maximum value of the image pixel intensity, and MSE denotes the mean squared error.

The closer the SSIM value approaches 1, the better the visual structural consistency between the restored image and the reference image, as well as the higher the perceptual matching level. SSIM is defined as:


$$\begin{array}{c} \text{SSIM}\left(x,y\right)=\frac{\left(2u_{x}u_{y}+C_{\text{1}}\right)\left(2\sigma_{xy}+C_{\text{2}}\right)}{\left(u_{x}^{\text{2}}+u_{y}^{\text{2}}+C_{\text{1}}\right)\left(\sigma_{x}^{\text{2}}+\sigma_{y}^{\text{2}}+C_{\text{2}}\right)} \end{array}$$ (15)

where u is the mean value, $\sigma_{xy}$ is the covariance, $\sigma^{\text{2}}$ is the variance, and $C_{\text{1}}$ and $C_{\text{2}}$ are constants.

CIEDE is used to measure the degree of color difference between the original image and the hazy image, and CIEDE can be expressed as:


$$\begin{array}{c} \text{CIEDE}=\;\sqrt{{\left(\frac{\Delta L^{\prime }}{K_{L}S_{L}}\right)}^{\text{2}}+{\left(\frac{\Delta C^{\prime }}{K_{C}S_{C}}\right)}^{\text{2}}+{\left(\frac{\Delta H^{\prime }}{K_{H}S_{H}}\right)}^{\text{2}}+R_{T}\left(\frac{\Delta C^{\prime }}{K_{C}S_{C}}\right)\left(\frac{\Delta H^{\prime }}{K_{H}S_{H}}\right)} \end{array}$$ (16)

where $\Delta L^{\prime }$, $\Delta C^{\prime }$, and $\Delta H^{\prime }$ are the lightness, chroma, and hue differences generated from the original image and the hazy image, respectively. $K_{L}$, $K_{C}$, and $K_{H}$ are three custom constant parameters. $S_{L}$, $S_{C}$, and $S_{H}$ are three weighting functions. $R_{T}$ is the rotation function that weights the hue and chroma differences.

### 4.3 Comparative experiments

In the comparative experiments, we compared the proposed DEMANet with other different methods, including prior-based methods (DCP [8], CAP [10], IHDCP [12]) and deep learning-based methods (AOD-Net [15], DehazeFormer [20], MixDehazeNet [17], DEA-Net [5]). For the deep learning-based methods, we used the same training and test datasets and retrained them with the source code provided by the authors. To ensure the fairness of quantitative comparisons, we adopt a unified evaluation script to calculate the PSNR, SSIM, and CIEDE metrics for all dehazed images generated by the proposed method and other dehazing algorithms. Experimental results are given in Table 1.

**Table 1:** Experimental results of image dehazing on the SateHaze1k and RRSHID datasets. Bold and underlined values represent the optimal and suboptimal results, respectively.

Methods	Haze1k-thin			Haze1k-moderate			Haze1k-thick			RRSHID-moderate		
	PSNR	SSIM	CIEDE	PSNR	SSIM	CIEDE	PSNR	SSIM	CIEDE	PSNR	SSIM	CIEDE
DCP	14.54	0.7309	10.7453	16.94	0.8401	8.8863	12.52	0.5895	15.1640	13.48	0.4155	23.8877
CAP	19.29	0.8522	8.8884	19.06	0.8726	9.5029	12.22	0.6393	18.0189	10.71	0.4359	30.2181
AOD-Net	18.19	0.8503	9.5634	18.48	0.8732	13.0099	15.17	0.7273	14.1764	18.86	0.5357	13.1019
DehazeFormer	24.34	0.9070	5.0319	**26.10**	0.9407	7.6287	21.79	0.8318	**6.7503**	23.21	0.6731	6.1007
MixDehazeNet	24.31	0.9070	5.4695	25.36	0.9255	7.4610	22.03	0.8366	6.9647	23.29	0.6551	6.4359
DEA-Net	24.88	0.9056	5.8278	25.68	0.9206	**6.8844**	22.26	0.8351	7.5569	23.26	0.6364	6.7230
IHDCP	19.17	0.8686	8.8039	17.22	0.8812	11.1749	11.85	0.6565	18.2753	6.35	0.3182	48.1403
ours	**25.29**	**0.9195**	**4.7869**	25.53	**0.9415**	7.5943	**22.49**	**0.8533**	6.9850	**23.53**	**0.6938**	**5.9185**

As can be seen from Table 1, conventional prior-based approaches (DCP, CAP, IHDCP) demonstrate poor results on all test datasets, especially on the Haze1k-thick and RRSHID-moderate datasets, showing obvious limitations. Deep learning-based methods (AOD-Net, DehazeFormer, MixDehazeNet, DEA-Net, ours) are significantly superior to traditional prior-based methods. Among them, as an early deep learning dehazing model, AOD-Net outperforms traditional prior-based methods but still has large room for improvement compared with recent network models. We conducted dehazing experiments using recent deep learning methods DehazeFormer, MixDehazeNet, and DEA-Net, and the results indicate that all three techniques perform comparably and yield satisfactory haze removal outcomes. When evaluated on the SateHaze1k-Thin and RRSHID-moderate dataset, our approach comes out on top across all performance metrics.

**Fig 6:**
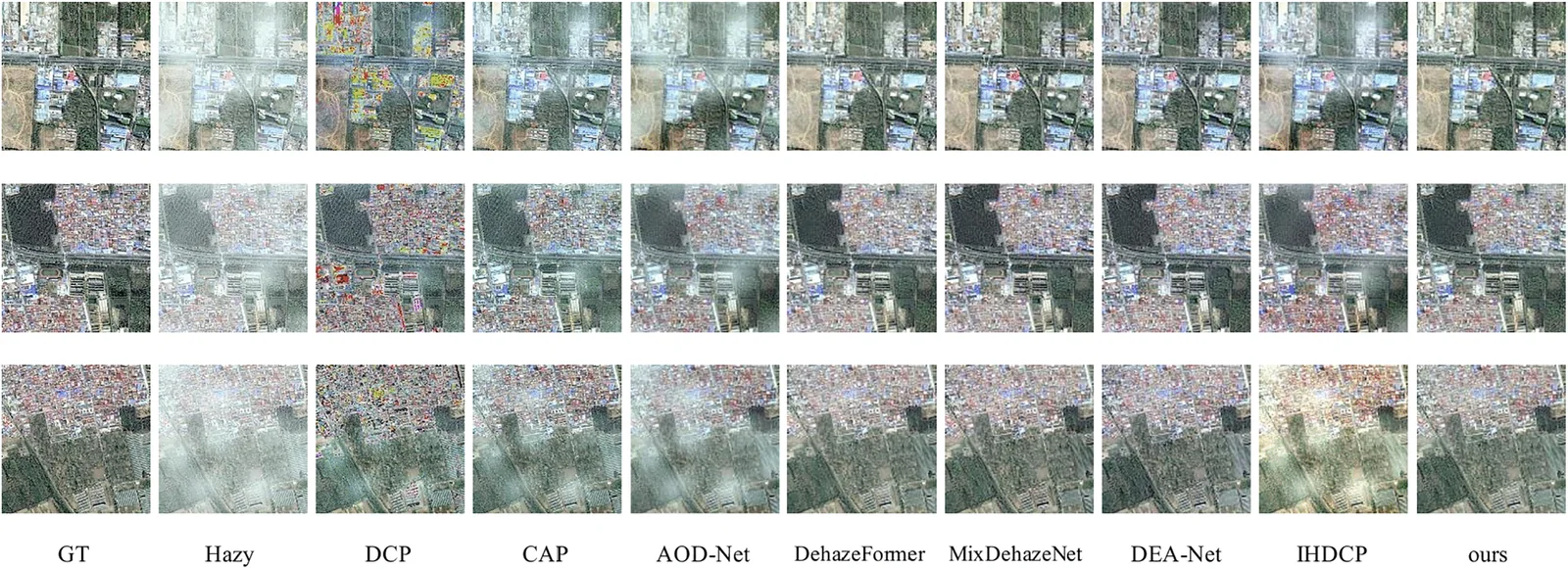
Visual comparison results of the StateHaze1k-thin dataset.

**Fig 7:**
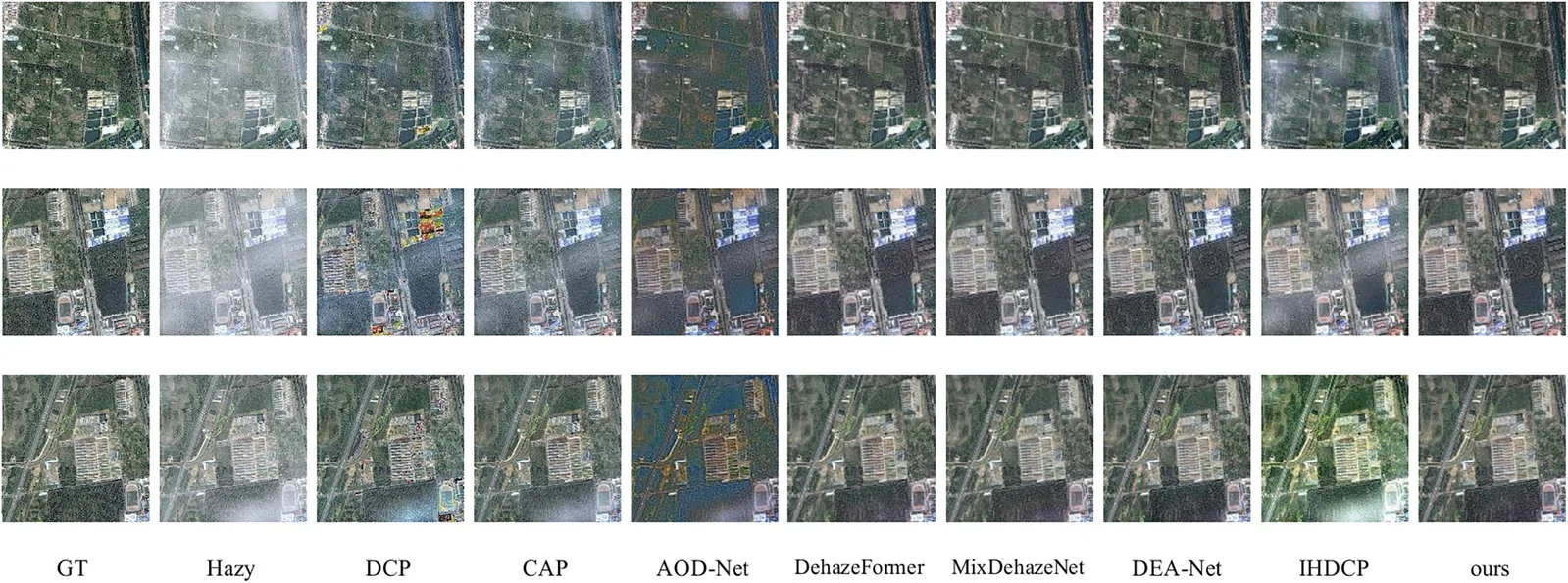
Visual comparison results of the StateHaze1k-moderate dataset.

**Fig 8:**
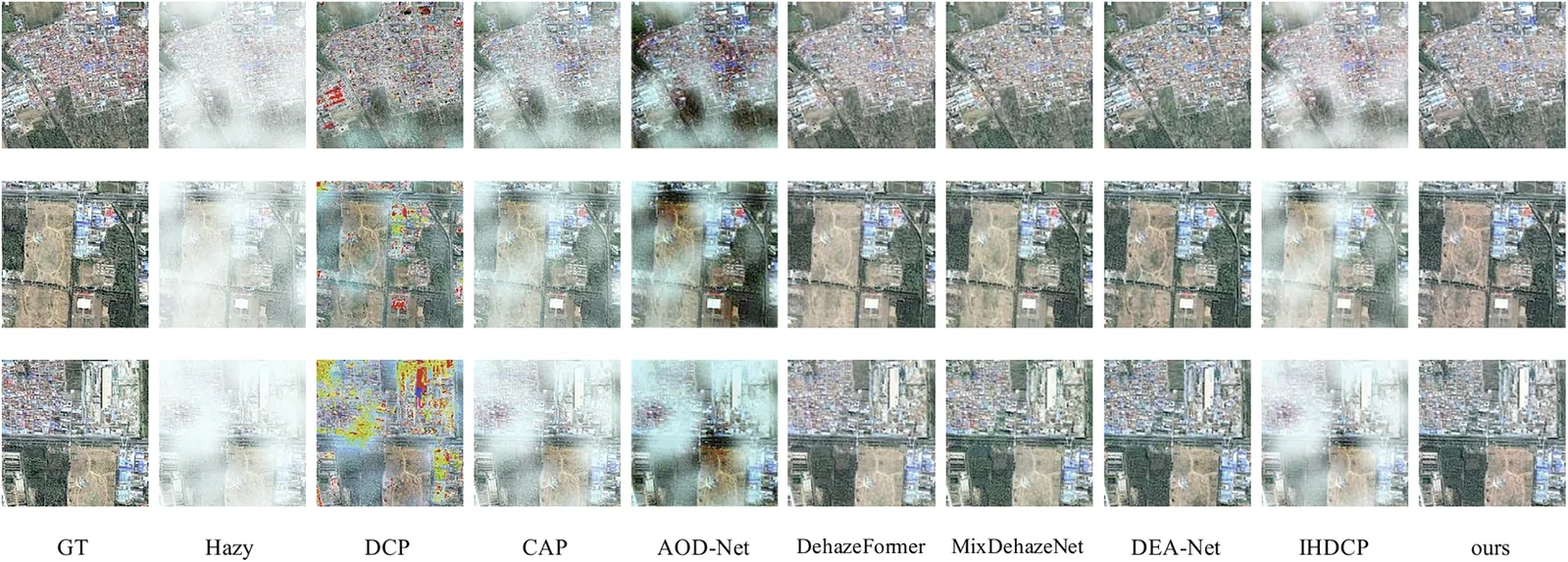
Visual comparison results of the StateHaze1k-thick dataset.

**Fig 9:**
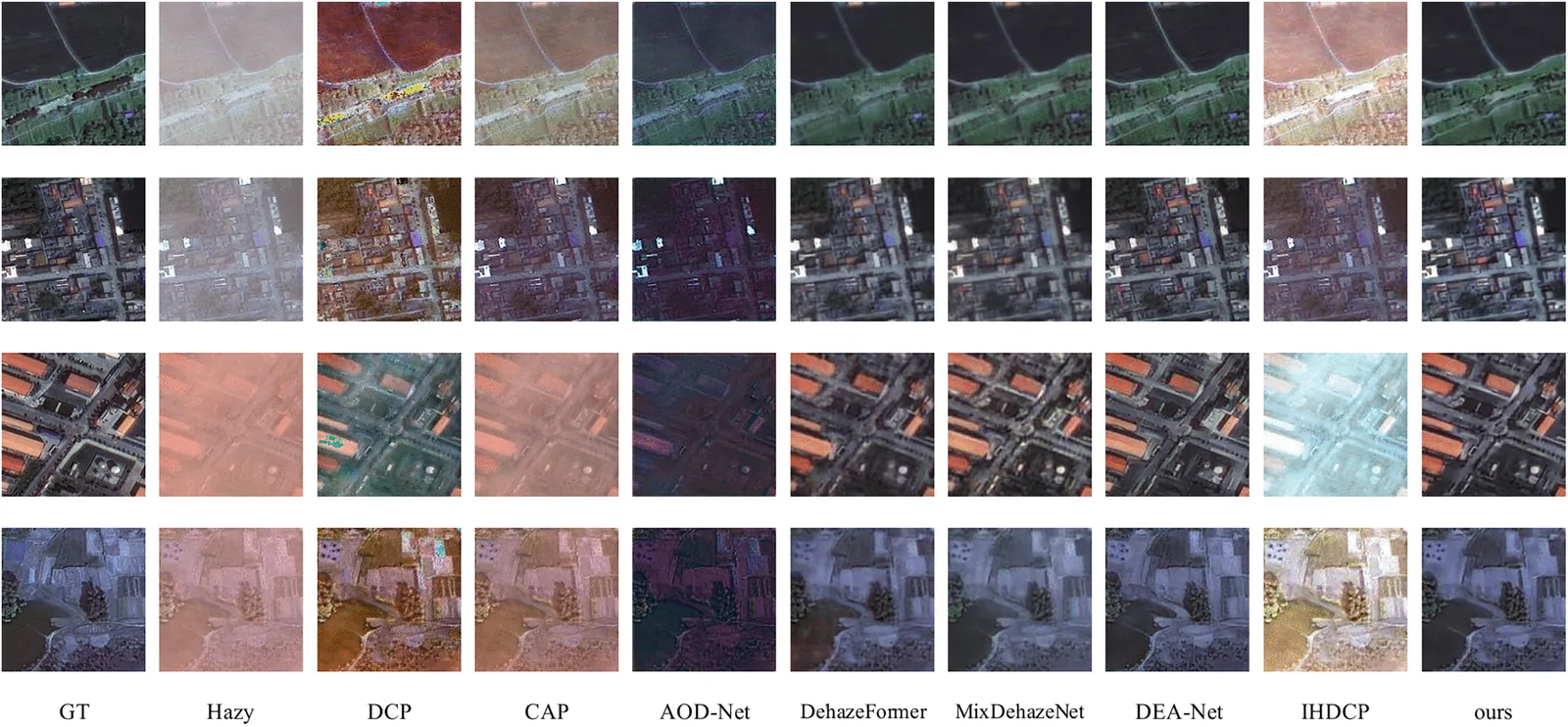
Visual comparison results of the RRSHID-moderate dataset.

Figs 6–9 show the visualization results compared with different methods on the SateHaze1k-thin, SateHaze1k-moderate, SateHaze1k-thick and RRSHID-moderate datasets, respectively.

According to the visual comparison results on the four datasets including SateHaze1k-thin, SateHaze1k-moderate, SateHaze1k-thick and RRSHID-moderate, traditional prior-based methods show unsatisfactory dehazing effects. Among them, the DCP and CAP methods suffer from severe color distortion after dehazing. In thin haze scenarios of the SateHaze1k dataset a slight amount of haze still persists, and they cannot effectively penetrate the haze in thick haze scenes. On the RRSHID-moderate dataset, these methods exhibit problems such as unrecoverable colors, blurred details, and lost textures, making it difficult to restore key information in RSI. Among the deep learning-based methods, AOD-Net shows limited dehazing performance, with obvious haze residue and blurred edges after processing. Although DehazeFormer, MixDehazeNet, and DEA-Net can effectively eliminate most haze, they still have deficiencies in complex scenarios. On the SateHaze1k-thick dataset, local details are slightly blurred and the colors deviate from the ground truth (GT). On the RRSHID-moderate dataset, slight color fading is observed. Experimental results across all dehazed images demonstrate that our method yields outputs much closer to the ground truth in thin, moderate, and thick haze scenarios. It can not only completely remove haze but also effectively restore the texture details and original colors of images without obvious blurring, color distortion, or detail loss. The visual effects are comprehensively better than all comparison methods, demonstrating that the proposed method has distinct advantages in both dehazing performance and detail preservation.

### 4.4 Ablation experiment

**Fig 10:**
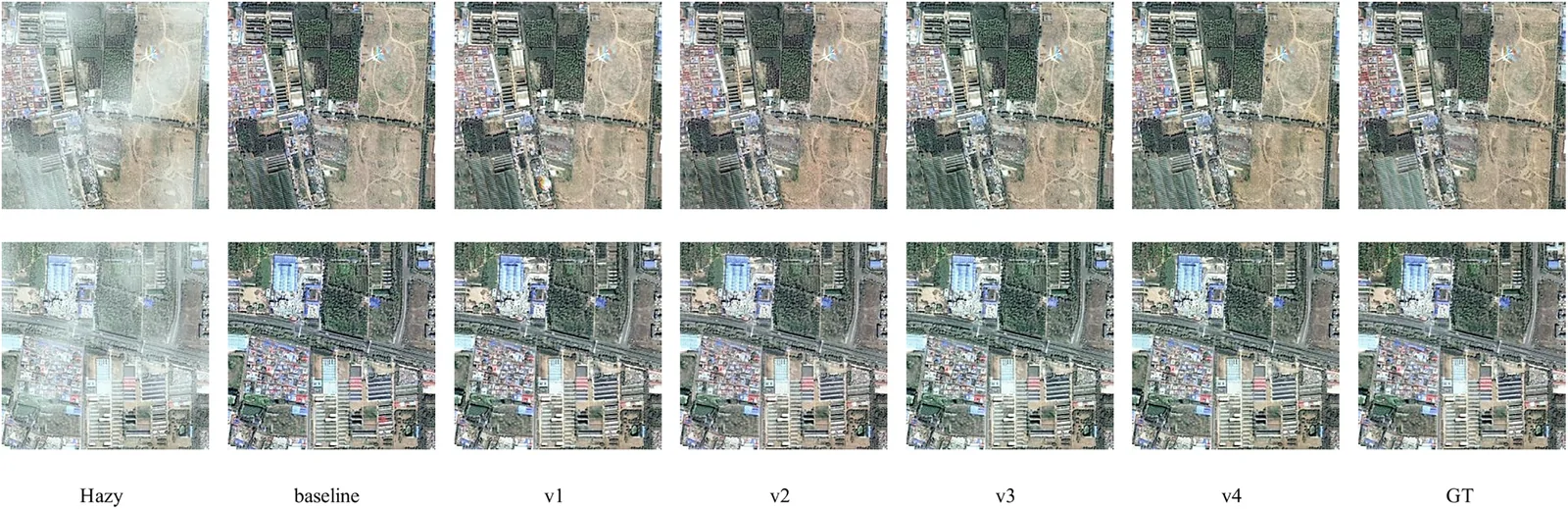
Comparison of ablation experiment results.

Ablation testing of the method's individual components confirms the core module's efficacy and training strategy. The corresponding experimental results are listed in Table 2. In the experiments, the Basic Residual Block (BasicResidualBlock) [27] and the CGAFusion module are used as the baseline model. v1 is based on the baseline model, where the L1 loss is replaced by an adaptive weighted fusion loss based on heteroscedastic uncertainty, and combined with contrastive loss for model training. v2 first introduces the FIB module to replace the CGAFusion module in the baseline model. Experimental outcomes illustrate that the performance of the model is greatly enhanced, proving that FIB can fuse multi-scale features more efficiently and alleviate the problem of feature loss in the dehazing task. v3 further introduces DAB on the basis of v2 to replace the basic residual block of Level 3 in the deep feature processing layer of the network. Embedding the DAB module strengthens the model’s capability of capturing image details and further elevates the evaluation metrics. v4 fully introduces RDPB on the basis of the module combination of v3 to replace all basic residual blocks of Level 1 and Level 2 in the shallow and middle feature processing layers of the network, completing the integration of all innovative modules proposed in this paper. At this time, the model achieves optimal performance, reflecting the synergy and complementarity of each innovative module. The experimental results of different ablation schemes are shown in Fig 10.

**Table 2:** Ablation experiment results on the SateHaze1k-thin dataset.

StateHaze1k-thin					
Method	baseline	v1	v2	v3	v4
${ℒ}_{UMS}$		✓	✓	✓	✓
FIB			✓	✓	✓
DAB				✓	✓
RDPB					✓
PSNR	23.73	24.03	24.70	24.84	**25.29**
SSIM	0.9001	0.9133	0.9149	0.9169	**0.9195**
CIEDE	6.3908	5.1404	5.2287	5.1770	**4.7869**

## 5 Conclusion

This paper proposes a Dehazing Enhanced Multi-branch Attention Network (DEMANet) suitable for remote sensing image dehazing. It adopts a U-Net-like encoder-decoder architecture combined with innovative module designs at multiple feature levels, which effectively solves the common problems in remote sensing image dehazing, such as detail loss, inaccurate haze region location, insufficient global semantic information mining, and feature redundancy. Specifically, DEMANet introduces the Residual Dual Path Block (RDPB) in the shallow and middle feature extraction stage, achieving dual optimization of detail preservation and feature redundancy reduction. For cross-stage feature interaction, the Feature Interaction Block (FIB) adopts a haze-guided mechanism combined with an attention mechanism, enabling precise alignment and efficient fusion of cross-stage features. The Dual Attention Block (DAB) is embedded in deep feature processing, further enhancing the ability to capture global information in complex haze regions. Experimental results on two public remote sensing dehazing datasets, SateHaze1k and RRSHID, show that compared with current mainstream advanced dehazing methods, DEMANet can not only remove haze from remote sensing images more accurately and solve the problem of uneven haze distribution, but also preserve detailed information and color authenticity of images to the greatest extent.

Although the proposed method has achieved promising dehazing performance for remote sensing images, there is still room for improvement. For instance, under complex and extreme haze conditions, the model lacks precision in color restoration, and its adaptability to complex haze conditions needs further enhancement. We will further expand the application scope of this work in the future and explore its feasibility in multiple severe weather removal tasks. In addition, since all datasets used in this paper are synthetic hazy data, they cannot fully reflect the complex interferences, haze distributions, and imaging distortions in real hazy environments. Follow-up work will focus on the difficulties of dehazing in real hazy scenes to construct an algorithm scheme suitable for real scenarios.

## References

[1] Dong W, Wang C, Xu X. ICL-Net: Inverse Cognitive Learning Network for Remote Sensing Image Dehazing. IEEE J Sel Top Appl Earth Observations Remote Sensing. 2024;17:16180–91. doi: 10.1109/jstars.2024.3454754

[2] Zhang X, Wang T, Luo W, Huang P. Multi-Level Fusion and Attention-Guided CNN for Image Dehazing. IEEE Transactions on Circuits and Systems for Video Technology. 2021;31(11):4162–73. doi: 10.1109/TCSVT.2020.3046625

[3] Gui J, Cong X, Cao Y, Ren W, Zhang J, Zhang J i, et al. A Comprehensive Survey and Taxonomy on Single Image Dehazing Based on Deep Learning. ACM Computing Surveys. 2023;55(13s):1–37. doi: 10.1145/3576918

[4] Kim N, Choi I-S, Han S-S, Jeong C-S. DA-Net: Dual Attention Network for Haze Removal in Remote Sensing Image. IEEE Access. 2024;12:136297–312. doi: 10.1109/access.2024.3459588

[5] Chen Z, He Z, Lu Z-M. DEA-Net: Single Image Dehazing Based on Detail-Enhanced Convolution and Content-Guided Attention. IEEE Trans Image Process. 2024;33:1002–15. doi: 10.1109/TIP.2024.3354108 38252568

[6] Li H, Li J, Wei H, Liu Z, Zhan Z, Ren Q. Slim-neck by GSConv: a lightweight-design for real-time detector architectures. J Real-Time Image Proc. 2024;21(3). doi: 10.1007/s11554-024-01436-6

[7] Ju M, Gu Z, Zhang D. Single image haze removal based on the improved atmospheric scattering model. Neurocomputing. 2017;260:180–91. doi: 10.1016/j.neucom.2017.04.034

[8] He K, Sun J, Tang X. Single Image Haze Removal Using Dark Channel Prior. IEEE Trans Pattern Anal Mach Intell. 2011;33(12):2341–53. doi: 10.1109/TPAMI.2010.168 20820075

[9] Fattal R. Dehazing Using Color-Lines. ACM Trans Graph. 2014;34(1):1–14. doi: 10.1145/2651362

[10] Zhu Q, Mai J, Shao L. A Fast Single Image Haze Removal Algorithm Using Color Attenuation Prior. IEEE Trans Image Process. 2015;24(11):3522–33. doi: 10.1109/TIP.2015.2446191 26099141

[11] Li P, Tian J, Tang Y, Wang G, Wu C. Deep Retinex Network for Single Image Dehazing. IEEE Trans Image Process. 2021;30:1100–15. doi: 10.1109/TIP.2020.3040075 33259301

[12] Liu Y, Li T, Tan C, Ren W, Ancuti C, Lin W. IHDCP: Single Image Dehazing Using Inverted Haze Density Correction Prior. IEEE Trans Image Process. 2026;35:1448–61. doi: 10.1109/TIP.2026.3657636 41610348

[13] Cai B, Xu X, Jia K, Qing C, Tao D. DehazeNet: An End-to-End System for Single Image Haze Removal. IEEE Trans Image Process. 2016;25(11):5187–98. doi: 10.1109/TIP.2016.2598681 28873058

[14] Ren W, Pan J, Zhang H, Cao X, Yang M-H. Single Image Dehazing via Multi-scale Convolutional Neural Networks with Holistic Edges. Int J Comput Vis. 2019;128(1):240–59. doi: 10.1007/s11263-019-01235-8

[15] Li B, Peng X, Wang Z, Xu J, Feng D. AOD-Net: All-in-One Dehazing Network. In: 2017 IEEE International Conference on Computer Vision (ICCV), 2017. 4780–8. 10.1109/iccv.2017.511

[16] Engin D, Genc A, Ekenel HK. Cycle-Dehaze: Enhanced CycleGAN for Single Image Dehazing. In: 2018 IEEE/CVF Conference on Computer Vision and Pattern Recognition Workshops (CVPRW), 2018. 938–9388. 10.1109/cvprw.2018.00127

[17] Lu L, Xiong Q, Xu B, Chu D. MixDehazeNet: Mix Structure Block For Image Dehazing Network. In: 2024 International Joint Conference on Neural Networks (IJCNN), 2024. 1–10. 10.1109/ijcnn60899.2024.10651326

[18] Wen Y, Gao T, Li Z, Zhang J, Chen T. Encoder-Minimal and Decoder-Minimal Framework for Remote Sensing Image Dehazing. In: ICASSP 2024 - 2024 IEEE International Conference on Acoustics, Speech and Signal Processing (ICASSP), 2024. 36–40. 10.1109/icassp48485.2024.10446125

[19] Qiu Y, Zhang K, Wang C, Luo W, Li H, Jin Z. MB-TaylorFormer: Multi-branch Efficient Transformer Expanded by Taylor Formula for Image Dehazing. In: 2023 IEEE/CVF International Conference on Computer Vision (ICCV), 2023. 12756–67. 10.1109/iccv51070.2023.01176

[20] Song Y, He Z, Qian H, Du X. Vision Transformers for Single Image Dehazing. IEEE Trans Image Process. 2023;32:1927–41. doi: 10.1109/TIP.2023.3256763 37030760

[21] Liu Y, Yan Z, Chen S, Ye T, Ren W, Chen E. NightHazeFormer: Single Nighttime Haze Removal Using Prior Query Transformer. In: Proceedings of the 31st ACM International Conference on Multimedia, 2023. 4119–28. 10.1145/3581783.3611744

[22] Liu Y, Wang X, Hu E, Wang A, Shiri B, Lin W. VNDHR: Variational Single Nighttime Image Dehazing for Enhancing Visibility in Intelligent Transportation Systems via Hybrid Regularization. IEEE Trans Intell Transport Syst. 2025;26(7):10189–203. doi: 10.1109/tits.2025.3550267

[23] Huang B, Li Z, Yang C, Sun F, Song Y. Single Satellite Optical Imagery Dehazing using SAR Image Prior Based on conditional Generative Adversarial Networks. In: 2020 IEEE Winter Conference on Applications of Computer Vision (WACV), 2020. 1795–802. 10.1109/wacv45572.2020.9093471

[24] Zhu Z-H, Lu W, Chen S-B, Ding CHQ, Tang J, Luo B. Real-World Remote Sensing Image Dehazing: Benchmark and Baseline. IEEE Trans Geosci Remote Sensing. 2025;63:1–14. doi: 10.1109/tgrs.2025.3584234

[25] Kingma DP, Ba J. Adam: A method for stochastic optimization. In: 2014. https://arxiv.org/abs/1412.6980

[26] He T, Zhang Z, Zhang H, Zhang Z, Xie J, Li M. Bag of Tricks for Image Classification with Convolutional Neural Networks. In: 2019 IEEE/CVF Conference on Computer Vision and Pattern Recognition (CVPR), 2019. 558–67. 10.1109/cvpr.2019.00065

[27] He K, Zhang X, Ren S, Sun J. Deep Residual Learning for Image Recognition. In: 2016 IEEE Conference on Computer Vision and Pattern Recognition (CVPR), 2016. 770–8. 10.1109/cvpr.2016.90

